# Alternative Splicing Analysis Reveals Adrenergic Signaling as a Novel Target for Protein Arginine Methyltransferase 5 (PRMT5) in the Heart

**DOI:** 10.3390/ijms26052301

**Published:** 2025-03-05

**Authors:** Shouye Jiao, Yimeng Zhang, Xiao Yang, Jian Wang, Zhenhua Li

**Affiliations:** 1 Department of Cell Biology, School of Basic Medicine, Qingdao University, Qingdao 266071, China; jsy15898753375@163.com (S.J.); 15689725912@163.com (Y.Z.); 2 State Key Laboratory of Medical Proteomics, Beijing Proteome Research Center, National Center for Protein Sciences, Beijing Institute of Lifeomics, Beijing 102206, China; yangx@bmi.ac.cn

**Keywords:** PRMT5, alternative splicing, adrenergic signaling, heart disease

## Abstract

Adrenergic signaling is critical for maintaining cardiac function and works by regulating heart rate, contractility, and stress responses. Protein arginine methyltransferase 5 (PRMT5), a key enzyme involved in gene expression, signal transduction, and RNA processing, has been revealed to be an important factor in heart disease. However, its specific effects on adrenergic signaling have not been fully elucidated. In this study, we examined the role of PRMT5 in the heart by analyzing alternative splicing events in cardiac tissues from *Prmt5*-deficient mice. High-throughput RNA sequencing and bioinformatics analyses identified significant alterations in alternative splicing, particularly in genes related to adrenergic signaling, which were further validated using reverse transcription PCR. These results underscore the role of PRMT5 as an important regulator of alternative splicing in the heart and identify adrenergic signaling as a novel target. Collectively, our findings offer new insights into the molecular mechanisms underlying cardiac function and suggest that PRMT5 is a potential therapeutic target for heart diseases.

## 1. Introduction

The adrenergic signaling pathway in the heart is essential for maintaining cardiovascular health. Activation of the adrenergic signaling pathway initiates a cascade of intracellular events, including the elevation of cyclic adenosine monophosphate (cAMP) levels and the subsequent activation of protein kinase A (PKA) [1]. The dysregulation of adrenergic signaling is implicated in heart failure and other cardiac pathologies, where prolonged sympathetic nervous system overactivation leads to receptor desensitization, impaired signal transduction, and reduced cardiac function [2,3]. Understanding the mechanisms that regulate β-adrenergic signaling is vital for developing therapeutic strategies to prevent or treat these conditions.

Alternative splicing is a vital post-transcriptional mechanism that broadens proteome diversity, allowing cells to adapt to varying physiological demands [4]. In the heart, alternative splicing governs critical developmental processes and maintains functional homeostasis [5]. Several splicing factors—including RNA-binding motif proteins—have been identified as the key regulators of splicing events that preserve sarcomere integrity, calcium signaling, and myocardial contraction [6]. Dysfunction in these splicing regulators leads to the mis-splicing of key genes, driving pathologies such as dilated cardiomyopathy (DCM), hypertrophic cardiomyopathy (HCM), and arrhythmias [7,8]. While advances in RNA sequencing technologies have revealed the presence of significant splicing alterations in cardiac diseases, their full scope and implications remain incompletely understood. Further exploration of the molecular mechanisms governing alternative splicing could provide new insights into the mechanisms underlying cardiac homeostasis.

As a major type II arginine methyltransferase, protein arginine methyltransferase 5 (PRMT5) includes an N-terminal TIM barrel domain that allows it to form a heterooctameric complex with MEP50, a middle Rossman fold domain responsible for methyltransferase activity, and a C-terminal β-barrel domain that supports dimerization to create the inner core tetramer [9,10]. PRMT5 symmetrically dimethylates the arginine residues on a wide range of substrates [11] and is extensively involved in the regulation of a variety of cellular physiological processes, such as RNA splicing, transcription, cell cycles, and DNA repair [12,13]. The dysregulation of PRMT5 is closely associated with the onset and progression of many diseases, including cancer [14,15,16], neurodegenerative diseases [17,18,19], cardiac hypertrophy [20,21,22], and so on. Emerging evidence also highlights its critical role in post-transcriptional mechanisms, particularly in RNA splicing, through interactions with the components of splicing machinery [23,24,25]. In the heart, PRMT5 regulates the RNA splicing of the O-GlcNAcase (*Oga*) gene, and its dysfunction results in elevated levels of protein O-GlcNAcylation, contributing to the development of dilated cardiomyopathy [26]. Despite these findings, the broader landscape of PRMT5′s regulatory role in alternative splicing in the heart is not well characterized, leaving critical gaps in our understanding of its contribution to cardiac homeostasis.

In this study, we performed bioinformatics analyses and identified significant splicing changes in *Prmt5*-deficient cardiac tissue. Experimental validation confirmed these findings, positioning PRMT5 as a key regulator of alternative splicing in the heart. Notably, we identified adrenergic signaling as a previously unrecognized target of PRMT5 regulation, which could substantially expand our understanding of the molecular mechanisms underlying the function of the heart. Given the critical role of adrenergic signaling in heart rate and contractility, our findings provide novel insights into cardiac molecular mechanisms and suggest that PRMT5 is a promising therapeutic target for heart disease.

## 2. Results

### 2.1. Analysis of Alternative Splicing in Cardiomyocyte-Specific Prmt5 Knockout Mice

To investigate the regulatory role of PRMT5 in alternative splicing in the heart, RNA sequencing (RNA-Seq) data from *Prmt5*-cKO (αMHC-Cre;*Prmt5*^fl/fl^) and control (*Prmt5*^fl/fl^) mice were reanalyzed. FASTQ sequencing reads were aligned to the mouse reference genome (mm10) using Hisat2, and the resulting BAM files were subjected to downstream analyses.

**Figure 1:**
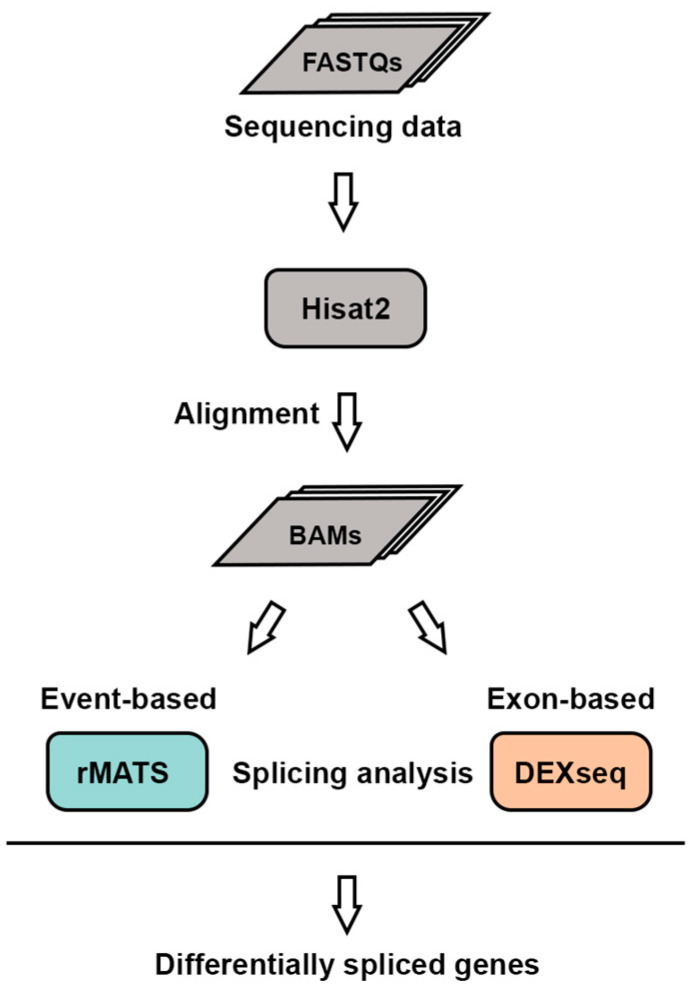
Workflow for alternative splicing analysis. RNA-seq datasets in FASTQ format were aligned to the mouse genome (mm10) using Hisat2, generating BAM files. Differentially spliced genes (DSGs) were identified using rMATS and DEXSeq.

Current methods for analyzing alternative splicing are often limited by high false positive rates, as most tools focus on a single category, such as isoform-, exon/intron-, or event-based analyses. This lack of comprehensive evaluation can compromise the reliability of splicing profiles. To address this limitation, we employed two complementary approaches—rMATS, an event-based method, and DEXSeq, an exon-based method. By combining the results from these different strategies, we identified differentially spliced genes with greater accuracy and minimized tool-specific biases (Figure 1).

### 2.2. Identification of Differentially Spliced Genes

**Figure 2:**
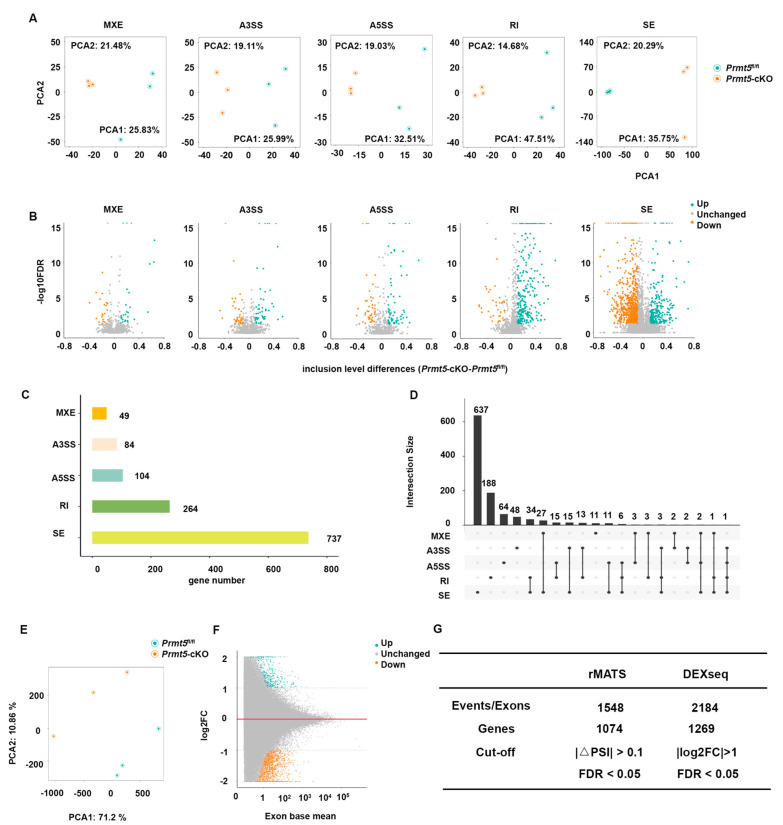
Differential splicing analysis of *Prmt5*-cKO hearts using rMATS and DEXSeq. (**A**) A principal component analysis (PCA) clearly separates the splicing profiles of the control and *Prmt5*-cKO groups, with each point representing an individual sample. (**B**) Volcano plot of differential splicing events identified by rMATS. Green dots represent significantly upregulated events and orange dots represent significantly downregulated events. (**C**) The number of differentially spliced genes seen across the five types of splicing event. (**D**) UpSet graph displaying the interactions between the five types of alternative splicing events and genes. (**E**) PCA plot of exon usage, demonstrating a tight grouping of the exon expression profiles by genotype, with each point representing a sample. (**F**) Volcano plot of differentially regulated exons identified by DEXseq. Green represents significantly upregulated exons, and orange represents significantly downregulated exons. (**G**) Summary of differentially spliced genes identified by rMATS and DEXseq.

Using rMATS, five types of splicing events were identified in the hearts of *Prmt5*-cKO and control mice: mutually exclusive exons (MXEs), alternative 3′ splice sites (A3SSs), alternative 5′ splice sites (A5SSs), retained introns (RIs), and skipped exons (SEs). A principal component analysis (PCA) revealed distinct clustering between the *Prmt5*-cKO and control samples, highlighting the altered splicing patterns in *Prmt5*-deficient mice (Figure 2A). Significant splicing alterations were visualized via volcano plots, using thresholds of |ΔPSI| > 0.1 and FDR < 0.05 (Figure 2B). The differential splicing events were then counted at the gene level to better understand their broader biological significance (Figure 2C). SE and RI events accounted for the largest number of differentially spliced genes (Figure 2C). Furthermore, certain genes contained multiple types of splicing alterations, emphasizing the complexity of alternative splicing regulated by PRMT5 in the heart (Figure 2D). In total, rMATS identified 1074 differentially spliced genes that were associated with 1548 splicing events (Figure 2G).

Similarly, DEXSeq was used to analyze alternative splicing, focusing on exon-level usage differences between the *Prmt5*-cKO and control samples. The PCA of the DEXSeq results also showed a clear distinction between the two groups, consistent with the findings from rMATS (Figure 2E). Volcano plots were used to visualize the differentially upregulated and downregulated exons, further corroborating the significant impact of *Prmt5* deficiency on splicing regulation (Figure 2F). Using a filter of |log2FC| > 1 and an FDR < 0.05, DEXSeq identified 1269 differentially spliced genes involved in 2184 splicing events (Figure 2G).

These findings provide a comprehensive overview of how the alternative splicing landscape in cardiomyocytes is influenced by *Prmt5* knockout.

### 2.3. Functional Enrichment Analysis of Differentially Spliced Genes

**Figure 3:**
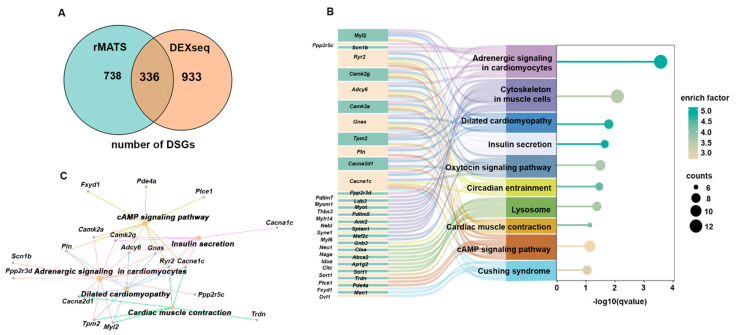
Functional enrichment analysis of differentially spliced genes. (**A**) Venn diagram showing the number of differentially spliced genes (DSGs) identified by rMATS and DEXseq. (**B**) A bubble plot combined with a Sankey diagram demonstrating the top 10 most significant KEGG pathways and the genes within each pathway on the *y*-axis and their statistical significance (−log10(q-value)) on the *x*-axis. (**C**) Network diagram of the differentially spliced genes and their associated KEGG pathways.

To investigate the impact of the splicing abnormalities caused by *Prmt5* knockout on cardiac function and signaling pathways, we focused on identifying the genes associated with the differentially spliced events recorded. By integrating the results from both the rMATS and DEXSeq analyses, we established which differentially spliced genes (DSGs) were identified by both methods, ensuring a high level of confidence in our findings (Figure 1). This integrative analysis revealed a total of 336 differentially spliced genes (Figure 3A). Following the identification of these genes, we conducted a KEGG pathway enrichment analysis to explore their potential functional implications. The analysis uncovered significant enrichment in pathways related to adrenergic signaling, dilated cardiomyopathy, myocardial contractility, cAMP signaling, and insulin secretion (Figure 3B). Among these, adrenergic signaling ranked at the top based on its enrichment factor and q-value (Figure 3B). A network analysis visualized the interactions between the DSGs and enriched pathways, emphasizing the direct involvement of adrenergic signaling-related DSGs in cardiac muscle contraction and dilated cardiomyopathy (Figure 3C).

These findings highlight the pivotal role of PRMT5 in regulating the alternative splicing of genes involved in β-adrenergic signaling and its broader implications for cardiac function.

### 2.4. Validation of DSGs in the Adrenergic Signaling Pathway

**Figure 4:**
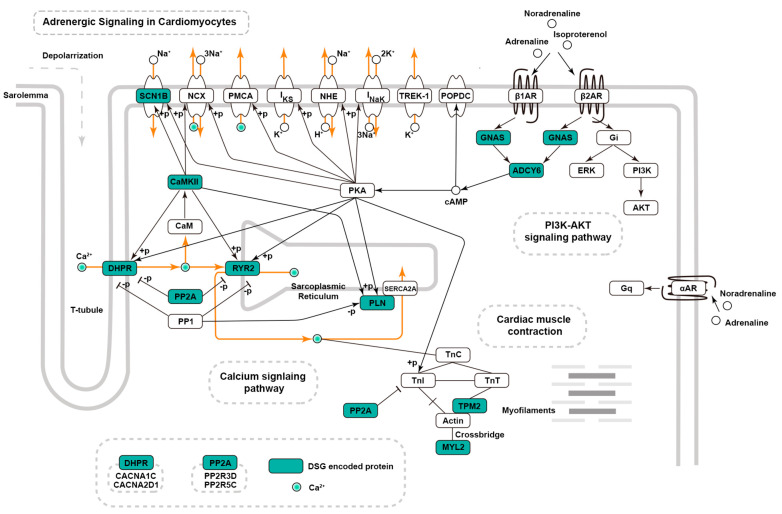
KEGG pathway map showing the differentially spliced genes within the adrenergic signaling pathway in the heart. Differentially spliced genes are colored green.

The adrenergic signaling pathway plays a central role in cardiac excitation–contraction coupling [27]. The activation of adrenergic receptors stimulates downstream signaling pathways, such as the cAMP-PKA signaling cascade, which are essential for calcium handling and myocardial contractility in the heart [28,29,30]. The absence of PRMT5 leads to widespread abnormal alternative splicing of genes associated with the adrenergic signaling pathway (Figure 4). We then validated these DSGs by categorizing them into two groups: ion channel protein-coding genes (*Scn1b*, *Cacna2d1*, *Ryr2*) and non-channel protein-coding genes (*Gnas*, *Adcy6*, *Ppp2r5c*, *Ppp2r3d*, *Camk2α*, *Camk2g*).

**Figure 5:**
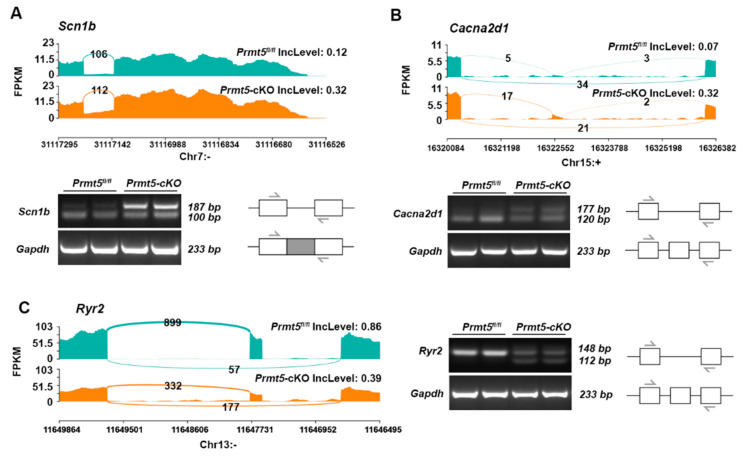
Validation of alternative splicing events in channel protein-coding genes of the adrenergic signaling pathway. Sashimi plots showing RNA-seq read densities along exons and junctions for the *Scn1b* (**A**), *Cacna2d1* (**B**), and *Ryr2* (**C**) genes in control (green) and *Prmt5*-cKO (orange) mice. Inclusion levels (IncLevels), calculated with rMATS, are shown. RT-PCR validation of the indicated splicing events in the hearts of control and *Prmt5*-cKO mice is shown at the bottom for *Scn1b* (**A**) and *Cacna2d1* (**B**) and to the right for *Ryr2* (**C**) of these plots. Arrows denote the positions of the primers used. The gray box represents the intron.

In terms of the ion channel protein-coding genes, *Scn1b* exhibited an increased level of RI, with an RT-PCR confirming a higher expression of intron retention isoforms in *Prmt5*-cKO mice (Figure 5A). *Cacna2d1* showed an increased level of exon inclusion, with an RT-PCR identifying a higher expression of exon-inclusion isoforms in *Prmt5*-cKO mice (Figure 5B). An SE event was also confirmed in *Ryr2*, which displayed decreased exon inclusion (Figure 5C).

**Figure 6:**
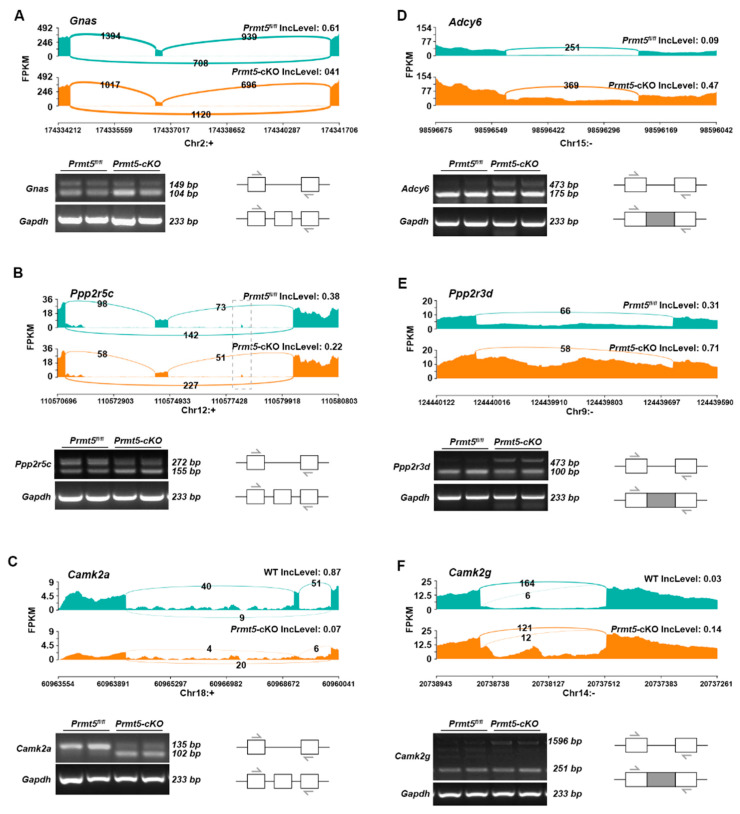
Validation of alternative splicing events in non-channel protein-coding genes of the adrenergic signaling pathway. Sashimi plots showing RNA-seq read densities along exons and junctions for each gene in the control (green) and *Prmt5*-cKO (orange) samples. IncLevels calculated with rMATS are shown. (**A**–**C**) Sashimi plots and RT-PCRs showing the SE events identified in *Gnas* (**A**), *Ppp2r5c* (**B**), and *Camk2a* (**C**). (**D**–**F**) Sashimi plots and RT-PCRs showing the RI events identified in *Adcy6* (**D**), *Ppp2r3d* (**E**), and *Camk2g* (**F**). Arrows denote the positions of the primers used. The gray boxes represents the introns.

For non-channel protein-coding genes, alternative splicing events were also validated through Sashimi plot visualizations and RT-PCR. Sashimi plots of *Gnas*, *Ppp2r5*c*,* and *Camk2α* revealed decreased levels of exon inclusions, with the RT-PCR confirming higher levels of exon-skipping isoforms (Figure 6A–C). RI events were identified in *Adcy6*, *Ppp2r3d*, and *Camk2g*, with the RT-PCR detecting increased levels of long fragments with retained introns (Figure 6B–F).

Collectively, these findings underscore the role of PRMT5 in modulating the alternative splicing of both channel and non-channel protein molecules in the β-adrenergic signaling pathway.

## 3. Discussion

Alternative splicing is a crucial regulatory process in the heart, ensuring the production of the diverse array of protein isoforms required for proper cardiac function during development and stress [31]. Numerous splicing factors, including RBM20, QKI, and RBPMS, have been shown to regulate critical genes involved in sarcomere assembly, calcium handling, and myocardial contractility. For instance, *Rbm20* knockout mice exhibit dilated cardiomyopathy (DCM) due to the disrupted splicing of genes such as *Titin* and *Camk2d*, impairing sarcomere structure and calcium signaling [32]. Similarly, Qki deletion impacts cardiomyocyte differentiation by misregulating genes essential for sarcomerogenesis and contractile function [33]. The splicing factor RBPMS modulates sarcomeric protein isoforms such as *Pdlim5* and *Ttn*, with its cardiac-specific knockout causing severe contractile defects and DCM in mice [34]. Expanding this paradigm, our study highlights PRMT5 as a significant regulator of alternative splicing in the heart. A loss of PRMT5 causes widespread splicing dysregulation that particularly affects β-adrenergic signaling genes, which are crucial for myocardial contractility. For instance, *Scn1b* encodes the β1 subunit of voltage-gated sodium channels, and its deletion disrupts calcium homeostasis, thereby increasing susceptibility to arrhythmias [35]. *Adcy6* regulates the response of the β-adrenergic receptor by catalyzing the generation of cAMP. The targeted deletion of *Adcy6* impairs cAMP production and calcium handling, leading to left ventricular dysfunction [36]. Additionally, as one of the regulatory subunit-coding genes of PP2A, *Ppp2r5c* suppresses excessive PP2A activity in an autoinhibitory manner. The knockout of *Ppp2r5c* in mice leads to increased PP2A activity, a decreased heart rate, and conduction defects [37]. These results position PRMT5 alongside other key splicing factors, underscoring its essential role in maintaining cardiac homeostasis. Given its regulatory influence over cardiac-specific splicing events, PRMT5 may represent a potential therapeutic target for treating cardiac dysfunctions linked to splicing abnormalities.

PRMT5 plays a highly conserved role in regulating RNA splicing across different cell types by symmetrically dimethylating spliceosomal proteins, ensuring accurate pre-mRNA processing. However, emerging evidence reveals that PRMT5 also exhibits tissue-specific regulatory effects, targeting distinct genes critical to the function of individual organs. In hematopoietic stem cells, a PRMT5 loss impairs the splicing of key genes involved in DNA repair and cell survival, triggering *P53*-mediated apoptosis and stem cell depletion [38]. In thymic epithelial cells, PRMT5 is essential for the splicing of key transcripts such as *Aire*, a regulator of immune tolerance, and several tissue-restricted antigens, with its deficiency resulting in autoimmune phenotypes [39]. In the heart, our previous work identified *Oga* as a key target of PRMT5, linking its splicing regulation to cardiac homeostasis and protection against dilated cardiomyopathy [26]. However, this understanding remains incomplete, as additional cardiac-specific targets and mechanisms are likely involved. Our findings further underscore the tissue-specific mechanisms by which PRMT5 governs β-adrenergic signaling pathways and myocardial function, but future research is needed to unravel the unique tissue-specific functions and pathways governed by PRMT5 in various physiological and pathological settings.

The regulation of adrenergic signaling occurs at multiple levels and involves complex mechanisms that influence both receptor expression and functionality. At the transcriptional level, the expression of the β2-adrenergic receptor is modulated by the cAMP response element (CRE) in its 5′ flanking region [40], which is recognized by the CRE binding protein (CREB), the activation of which enhances β2-adrenergic receptor transcription and leads to transient increases in steady-state mRNA levels. However, prolonged agonist exposure results in a downregulation of β2-adrenergic receptor expression due to reduced mRNA stability and the involvement of inhibitory factors like CREM [41]. Post-translational adrenergic signaling is primarily regulated by phosphorylation, with both β1- and β2-adrenergic receptors featuring phosphorylation sites targeted by kinases such as PKA and β-adrenergic receptor kinase [42]. Our research using PRMT5 knockout mice reveals significant splicing changes in key genes associated with this signaling pathway, highlighting the critical regulation of adrenergic signaling at the post-transcriptional level.

While this study provides valuable insights into the role of PRMT5 in regulating alternative splicing in the heart, there are several important limitations that should be considered. First, our findings are primarily based on a *Prmt5* knockout mouse model, so further validation is required to confirm the relevance of these findings in human disease. Additionally, while this study identifies significant changes in alternative splicing that are associated with PRMT5 loss, the physiological and pathological consequences of these aberrant splicing events need to be further investigated.

In summary, our findings provide new insights into the effects of PRMT5 on the adrenergic signaling pathway due to its regulatory role in splicing. This discovery could pave the way for identifying novel therapeutic targets for cardiomyopathy associated with PRMT5 deficiency. By targeting PRMT5 and its influence on alternative splicing in adrenergic signaling genes, therapeutic strategies may be developed to restore normal heart function and address cardiac dysfunctions linked to splicing abnormalities.

## 4. Materials and Methods

### 4.1. RNA Sequencing Data Processing

RNA sequencing data, in FASTQ format, from the heart tissues of *Prmt5*-cKO and *Prmt5*^fl/fl^ mice were obtained as previously described. Paired-end reads were aligned to the mouse reference genome (mm10) from the UCSC using the Hisat2 aligner (version 2.2.1) with its default settings. SAM files were converted to BAM files and indexed with samtools (version 1.20).

### 4.2. Differential Splicing Analysis

Differential exon usage was analyzed using DEXSeq (version 1.48.0). BAM files were processed with featureCounts (version 2.0.6) to quantify exon-level read counts. A reference annotation was generated using the Subread-to-DEXSeq Python script, https://github.com/vivekbhr/Subread_to_DEXSeq (accessed on 25 April 2024).

For alternative splicing events, rMATS (version 4.3.0) and maser (version 1.20.0) were used. The rMATS package identified five types of splicing events: alternative 5′ splice sites (A5SSs), alternative 3′ splice sites (A3SSs), skipped exons (SEs), retained introns (RIs), and mutually exclusive exons (MXEs). Visualizations were performed using maser and rmats2sashiplot (version 3.0.0).

### 4.3. Functional Enrichment Analysis

The pathway enrichment analysis was performed using the enrich KEGG function in the clusterProfiler package (version 4.11.1).

### 4.4. RT-PCR Validation

Total RNA was extracted from heart tissues using TRIzol (Invitrogen, Waltham, MA, USA). RNA (2000 ng) was reverse-transcribed to cDNA using a reverse transcription kit (TOYOBO, Ōsaka, Japan). Semi-quantitative PCR was performed using 2x Rapid Taq Master Mix (Vazyme, Nanjing, China), and the results were resolved on 2% agarose gels. The primer sequences used are listed in Supplementary Table S1.

### 4.5. Statistics

Significant splicing events were defined as having an average coverage > 5, |ΔPSI| > 0.1, and FDR < 0.05. Exons with a |log2(fold change)| > 1 and FDR < 0.05 were considered differentially used.

## Data Availability

Data is contained within the article and Supplementary Material.

## Supplementary Materials

The following supporting information can be downloaded at: https://www.mdpi.com/article/10.3390/ijms26052301/s1.
