# Supplementary material for: Alternative Splicing Analysis Reveals Adrenergic Signaling as a Novel Target for Protein Arginine Methyltransferase 5 (PRMT5) in the Heart
Source: Int J Mol Sci. 2025 Mar 5;26(5):2301. doi: 10.3390/ijms26052301 (PMC11899901; doi:10.3390/ijms26052301)
Supplement: Supplementary file 1 [file ijms-26-02301-s001.zip › ijms-3479744-supplementary.pdf]

Supplementary Table S1. List of primers used in this study.

Gene name	Primer sequence (5'->3')
RT-PCR	
Scn1b	Forward:ACCTGGCCATTACATCCGAG
	Reversed:TAGGGGCTGGCTCTTCCATG
Cacna2d1	Forward:CTCTGTCCCAATGGCTACTA
	Reversed:ACTCAGCATCGAGAAAATCC
Ryr2	Forward: TCAGACCCAGAGAGGACAGT
	Reversed:GTAGTTTGTGCCACACAGCT
Gnas	Forward: CCATTGTGAAGCAGATGAGG
	Reversed:TTCAATGGCCTCCTTCAGGT
Adcy6	Forward: GCCACCTACAACAGCTCAAT
	Reversed:AAGATGAAGGTCATGGCCAG
Ppp2r5c	Forward: GAAAGAGCGAGAAGAAGCGT
	Reversed: TGCAGTGAGCTTCCAAGGCT
Ppp2r3d	Forward: CGAAGGACTTCCACTCACGT
	Reversed:CTTAGCTCCTCACGCGAGAT
Camk2a	Forward:CTACTTTCTCTCCTCCACAG
	Reversed: TCTTCGTCCTCAATGGTGGT
Camk2g	Forward:ACTTTGAGAATCGTGAGTGG
	Reversed:TGAGCAGTGATAGTGGACAT
Gapdh	Forward:TGCCCAGAACATCATCCCT
	Reversed:GGTCCTCAGTGTAGCCCAAG
